# Spin-polarised electrons in a one-magnet-only Mott spin junction

**DOI:** 10.1038/s41598-017-13453-6

**Published:** 2017-10-16

**Authors:** L. De Pietro, G. Bertolini, Q. Peter, H. Cabrera, A. Vindigni, O. Gürlü, D. Pescia, U. Ramsperger

**Affiliations:** 1 Laboratorium fur Festkörperphysik, ETH Zürich, Zürich, 8093 Switzerland; 2 Department of Physics, Istanbul Technical University, Maslak, 34469 Istanbul Turkey

## Abstract

The current flowing through a Mott spin junction depends on the relative spin orientation of the two ferromagnetic layers comprising the “source” and “drain” sides of the junction. The resulting current asymmetry is detected as giant or tunnelling magnetoresistance depending on whether the two ferromagnets are separated by a metal or an insulator. Based on the fundamental principles of reciprocity for spin-dependent electron scattering, one can envisage a one-magnet-only spin junction in which the source is non-magnetic, and the spin information is encoded by the spin polarisation of the electrons that have crossed or are backscattered from the drain magnetic layer. The practical significance of using an unpolarised source is that the state of the magnetic layer can be modified without affecting the process of probing it. Whether this reciprocity is realised in the actual junctions is not yet known. Here, we demonstrate a nano-sized, one-magnet-only Mott spin junction by measuring the finite spin polarisation of the backscattered electrons. Based on this finding, we conclude that since the junction acts as a spin filter, the magnetic layer must experience a spin transfer that could become detectable in view of the high current densities achievable in this technology.

## Introduction

**Figure 1:**
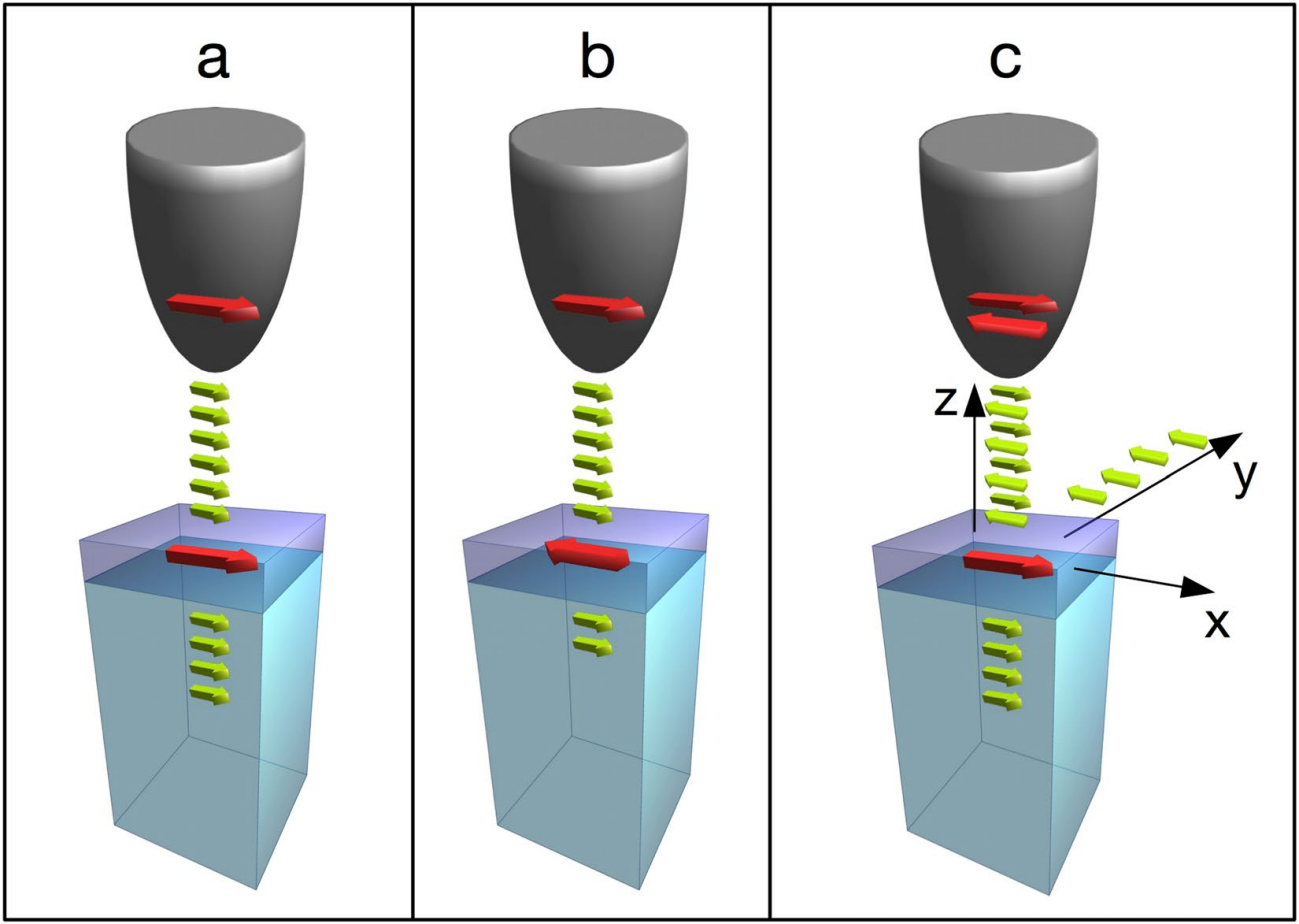
Different types of Mott spin junctions. The spin states of the source (top electrode) and drain are indicated by thick red arrows. The spin orientation of the propagating electrons is indicated with green arrows. The various spin states depicted in Fig. 1 are simplified to illustrate the principle of operation of the junctions. Generally, we expect that the spin state of the drain (red arrow) is also modified. In (**a**) (a two-magnet Mott spin junction), the spin state of the source (top electrode, red) is the same as the spin state of the drain (bottom electrode, red) and the current (green) flowing through the junction amounts to ${I}_{ \rightarrow }^{ \rightarrow }$. (**b**): When the spin state of the drain is reversed, a current asymmetry $\tfrac{{I}_{ \rightarrow }^{ \rightarrow }-{I}_{ \leftarrow }^{ \rightarrow }\,}{{I}_{ \rightarrow }^{ \rightarrow }+{I}_{ \leftarrow }^{ \rightarrow }}$ is obtained. (**c**) In the one-magnet-only Mott spin junction, the source electrode emits an equal number of electrons with the opposite spin state, but the exchange scattering^6^ with the spin state of the drain selects a well-defined spin state for both transmitted and backscattered electrons. The corresponding spin polarisation can be measured by spin polarimetry (for convenience, the coordinate system used in the paper is indicated).

In Mott spin junctions, the source is used to establish a reference spin state^1^. The spins of the drain electrode are then oriented parallel or antiparallel to the reference spin state, and the current flowing through the junction is modulated accordingly (Fig. 1a,b). The resulting current asymmetry is detected as a giant or tunnelling magnetoresistance depending on whether the two ferromagnets are separated by a metal or an insulator^1–4^. Suppose that we now replace the source with a non-magnetic material so that the reference spin state is unpolarised, i.e., the state consists of an equal number of spins pointing in two oppositely oriented directions (Fig. 1c). In this case, the switching of the spin state of the drain electrode does not produce any current asymmetry, but the same scattering mechanisms responsible for current asymmetry when a spin-polarised source is used are now filtering out the spins that have an unfavourable scattering cross-section, and the transmitted electrons are expected to become spin polarised^5,6,7^ (The spin polarisation vector of an electron ensemble is the quantum mechanical expectation value of the Pauli matrices. Given a certain direction $\overrightarrow{n}$ in space, the component ${P}_{\overrightarrow{n}}$ along $\overrightarrow{n}$ of the spin polarisation vector is given by ${\textstyle \tfrac{N(\uparrow )-N(\downarrow )}{N(\uparrow )+N(\downarrow )}}$, where N(↑)(N(↓)) is the number of electrons with spin parallel (antiparallel) to $\overrightarrow{n}$. ${P}_{\overrightarrow{n}}$ is a number between −1 (or −100%) and +1 (or +100%), these numbers representing fully spin polarised states. ${P}_{\overrightarrow{n}}=0$ labels an unpolarised ensemble of electrons. Because of the negative charge of the electron, the spin polarisation and the magnetization have opposite sign. In this paper, we prefer to plot the negative of ${P}_{\overrightarrow{n}}$ which is parallel to the magnetisation along $\overrightarrow{n}$). This principle of reciprocity is appealing because the encoded spin state can be detected without even changing the spin state of the drain. Moreover, when the spin state of the drain is altered by a controlled manipulation, the operation of detection of the spin state is not affected by the change. However, the spin signal is encoded in a quantity, namely, the spin polarisation of the electron system, that cannot be easily detected, especially if the electrons are buried within a junction.

The spin information is actually encoded in two further electronic systems, the backscattered electrons^8^ (We denote as “backscattered electrons” all electrons that are ejected by the primary source beam out of the target and succeed in escaping the junction. In the present experiment, we do not perform any energy analysis with the aim of selecting the kinetic energy of the electrons escaping the junction (see e.g. D. Zanin, ETH Zurich Diss. Nr. 24169, doi:10.3929/ethz-a-010866476 (2017))) (provided that they exist) and the spin state of the drain material itself. The backscattered electrons (Fig. 1c) could become spin polarised due to the spin-dependent scattering^6^. The drain material itself could change its spin polarisation – even if the injected beam is unpolarised – following the rule that the total angular momentum (orbital^1^ and spin components) for all the systems involved, e.g., injected electrons, transmitted electrons, backscattered electrons *and* drain electrons, must be conserved^2,7^. In this study, we access the spin state of the backscattered electrons in a situation in which the source consists of a non-magnetic W tip placed at the distance of between a few nm to a few tens of nm from the drain electrode made of ultrathin ferromagnetic Fe films grown epitaxially on a W (110) single crystal. At these distances, the direct quantum mechanical tunnelling between the source and drain that forms the basis for tunnelling magnetoresistance devices^3,4^ or scanning tunnelling microscopy (STM)^9–14^ is entirely suppressed, and the junction is therefore operated in the Fowler-Nordheim (or field emission) regime^14^, with vacuum separating the non-magnetic source from the magnetically active drain. In this regime, a system of backscattered electrons exists; in favourable circumstances, some of these electrons may propagate away from the junction^8,15–19^ and reach the macroscopic environment surrounding the junction for further processing, in this specific instance, for spin polarisation analysis using a spin polarimeter^20,21^ (Fig. 1c). We note that the opening of an STM junction to allow for backscattered electrons was introduced originally by R. Young^15^ approximately at the time when the first tunnelling magnetoresistance experiments were conducted^4^ and well before the discovery of STM^9^. A finite spin polarisation for the backscattered electrons was detected in 1989^16^, but the tip-target distance was 1 mm. Subsequently, in 1991, First *et al*.^17^ brought the tip to the range of distances *d* from the drain that are more appropriate for defining a junction (*d* ≈ 150 nm), but the spin signal was apparently lost.

With the aim of providing a rigorous proof-of-principle, several technical developments have been implemented with respect to previous works. The details of these developments can be found in the Methods section. First, we have established a reliable procedure for returning the STM tip to the Fowler-Nordheim regime while remaining at the same lateral *xy* coordinates^8^. This procedure allows us to find a relevant location by STM and to perform spin analysis while staying at this location. As a second step, the STM spectrometer is complemented with a spin polarimeter^20^ that accepts the backscattered electrons escaping the junction. Third, we have added the electron gun column of a commercial JEOL scanning electron microscope (JEOL Ltd, Akishima, Japan), typically providing a focused electron beam with several keV that also ejects backscattered electrons from the target into the same channel (along the *y*-direction in the present case, see Fig. 1c) used for the spin analysis of the backscattered electrons excited in the Fowler-Nordheim regime of STM. In this way, the as-yet-unknown spin sensitivity of the junction is gauged against a well-established experimental technique of spin analysis termed scanning electron microscopy with spin polarisation analysis (SEMPA)^22–26^. Finally, we have developed a procedure for eliminating the topographic cross-talk into the spin analysis that rendered a previous attempt at detecting the spin polarisation of the backscattered electrons inconclusive^17^. This procedure is performed by exploiting the property of any ferromagnetic sample that the graph of the magnetisation component *M* along the easy magnetisation axis as a function of the applied magnetic field *B*, i.e., the hysteresis loop, is closed and has a special symmetry: the average of *M*(*B*) over all values of *B* required to cover the entire hysteresis loop is exactly zero. Translated to the spin polarisation of the backscattered electrons, this symmetry defines a state of exactly zero-average spin polarisation that we use to eliminate any instrumental asymmetry not related to the sought-for spin polarisation from the spin detection.

The spin polarimeter detects two components, *P*_*x*_ and *P*_*z*_ (see Fig. 1c for the definition of the *x*- and *z-*coordinates) of the spin polarisation vector of the backscattered electrons. As explained in ref.^23^ the spin polarisation vector is directly proportional to the magnetisation vector of that region of the sample from which the backscattered electrons originate. The proportionality constant, however, is challenging to determine as it depends on kinematics parameters such as the energy of the primary electrons, the range of backscattered electron energies accepted by the spin polarimeter and the angle of acceptance. Accordingly, *P*_*x*_ and *P*_*y*_ provide reliable information regarding the relative magnitudes of the corresponding magnetisation components but not regarding their absolute values.

**Figure 2:**
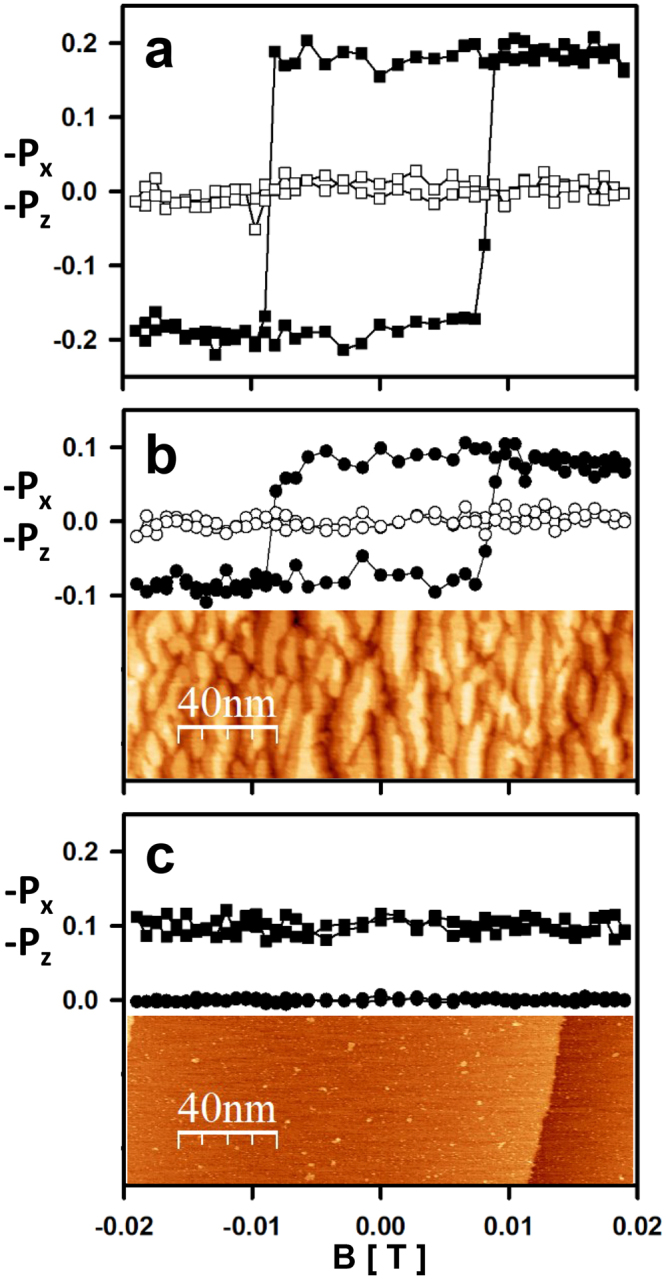
One-magnet-only Mott spin junction and evidence of spin polarisation of backscattered electrons. (**a**) −*P*_*x*_(*B*) (full squares) and −*P*_*z*_(*B*) (empty squares) measured with SEMPA (primary energy: 10 keV). The applied magnetic field (horizontal scale, common to a, b, and c and given in c) is given in units of Tesla. (**b**) −*P*_*x*_(*B*) (full circles) and −*P*_*z*_(*B*) (empty circles) measured in the Fowler-Nordheim regime of STM (d = 100 nm, primary energy: 62 eV, field emission current: ≈60 nA). Lower part: STM image (256 × 85 pixels) of a region of an 8-AL-thick Fe film covering a W (110) surface, on top of which the hysteresis curve is measured. (**c**) Full circles: −*P*_*x*_(*B*) on the Fe-uncovered surface measured in the Fowler-Nordheim regime of STM (d = 54 nm, primary energy: 71 eV, field emission current: ≈320 nA). Full squares: −*P*_*x*_(*B*) on the Fe-uncovered surface measured with SEMPA (shifted by +0.1 for clarity). Lower part: STM image (512 × 169 pixels) of the Fe-uncovered W (110) surface on top of which the spin polarisation is measured.

All experiments presented here are performed in ultra-high-vacuum conditions (i.e., pressure in the low 10^−10^ mbar range) and at room temperature. The target consists of a ferromagnetic Fe film several atomic layers (ALs) thick that is grown by molecular beam epitaxy on a non-magnetic W (110) single-crystal surface^8^. Such Fe films provide a stable reference ferromagnetic system^27^ at room temperature with a magnetisation that can be switched from one spatially uniform state along the easy in-plane [110]-direction (which is along the *x*-direction in the present coordinate system) to the spatially uniform state in the opposite direction. The switching is only possible when a sharply defined threshold magnetic field in the direction opposite to the magnetisation (called the coercive field) is applied. As a consequence, provided this field is not exceeded, the magnetic state achieved (for example) after a strong magnetic field pulse in one direction is maintained, even if the magnetic field is switched off. Technically, the phenomenon is described using the terms of remanent magnetisation and of a square hysteresis loop^27^. Figure 2a shows a typical hysteresis loop defining the magnetic state of the Fe film (8 ALs). The data in Fig. 2a are obtained using SEMPA (the technical aspects of applying a variable magnetic field *B* ^28^ and detecting the *P*(*B*) hysteresis loop are explained in the Methods section). The filled black squares correspond to the in-plane −*P*_*x*_ component and the empty black squares to the −*P*_*z*_ component of the polarisation vector, perpendicular to the film plane. As expected^27^, the −*P*_*x*_(*B*) graph shows an almost square hysteresis loop, and the −*P*_*z*_(*B*) component is undetectable within experimental accuracy. The coercive field (≈0.01 T) can be identified directly from Fig. 2a by locating the position along the horizontal axis at which −*P*_*x*_ switches sign. We note that the maximum value of −*P*_*x*_ (approximately 20%) is reduced with respect to the one expected from an Fe film in a SEMPA instrument^26^ (approximately 40%: for an explanation of this reduction, see the Methods section). Generally, we observe that the actual maximum value of −*P*_*x*_ depends on the set of voltages that we use to collect the backscattered electrons and cannot be assigned a quantitative significance yet – except that of being detectable or undetectable within experimental error. The same Fe film of Fig. 2a is approached at an arbitrary location for STM imaging (see the lower part of Fig. 2b) where the deposited Fe manifests its presence by the typical squamous topology^29^. Then, the tip is locked above a well-defined position within this squamous deposit and retracted to *d* ≈ 100 nm to obtain a hysteresis loop in the Fowler-Nordheim STM regime (Fig. 2b). Except for the maximum value of −*P*_*x*_ (approximately 10%), which is reduced with respect to the corresponding value in Fig. 2a, the hysteresis loops in Fig. 2b (x- and *z-*direction components) are identical to the ones recorded using SEMPA (Fig. 2a). The reduction of the maximum value of −*P*_*x*_ is expected from the dramatic lowering of the energy of the primary electrons, which is 62 eV in Fig. 2b, compared to 10 keV for the data obtained using SEMPA and shown in Fig. 2a ^26^. The time scale for the measurement of a single hysteresis loop in the Fowler-Nordheim regime is illustrated in the real-time movie provided as Additional Information. This time scale is in the 10 s range when using the present spin detector^21^.

The data shown in Fig. 2a and b are obtained under conditions in which only one side of the W (110) surface (with a total area of approximately 4 × 5 mm^2^) is exposed to Fe deposition, the Fe deposition on the other side being blocked by a thin silicon wafer placed almost in contact with the W surface (shadow mask deposition). When the tip is moved approximately 300 µm away from the Fe-covered side onto an Fe-uncovered position of the surface (see the STM image in the lower part of Fig. 2c), the hysteresis curve becomes undetectable within experimental accuracy (full circles in Fig. 2c). Also shown in Fig. 2c is −*P*_*x*_(*B*) measured on the Fe-uncovered side with SEMPA (black filled squares). Figure 2a–c demonstrate the spin polarisation of backscattered electrons in a Mott spin junction containing only one magnetically active electrode.

**Figure 3:**
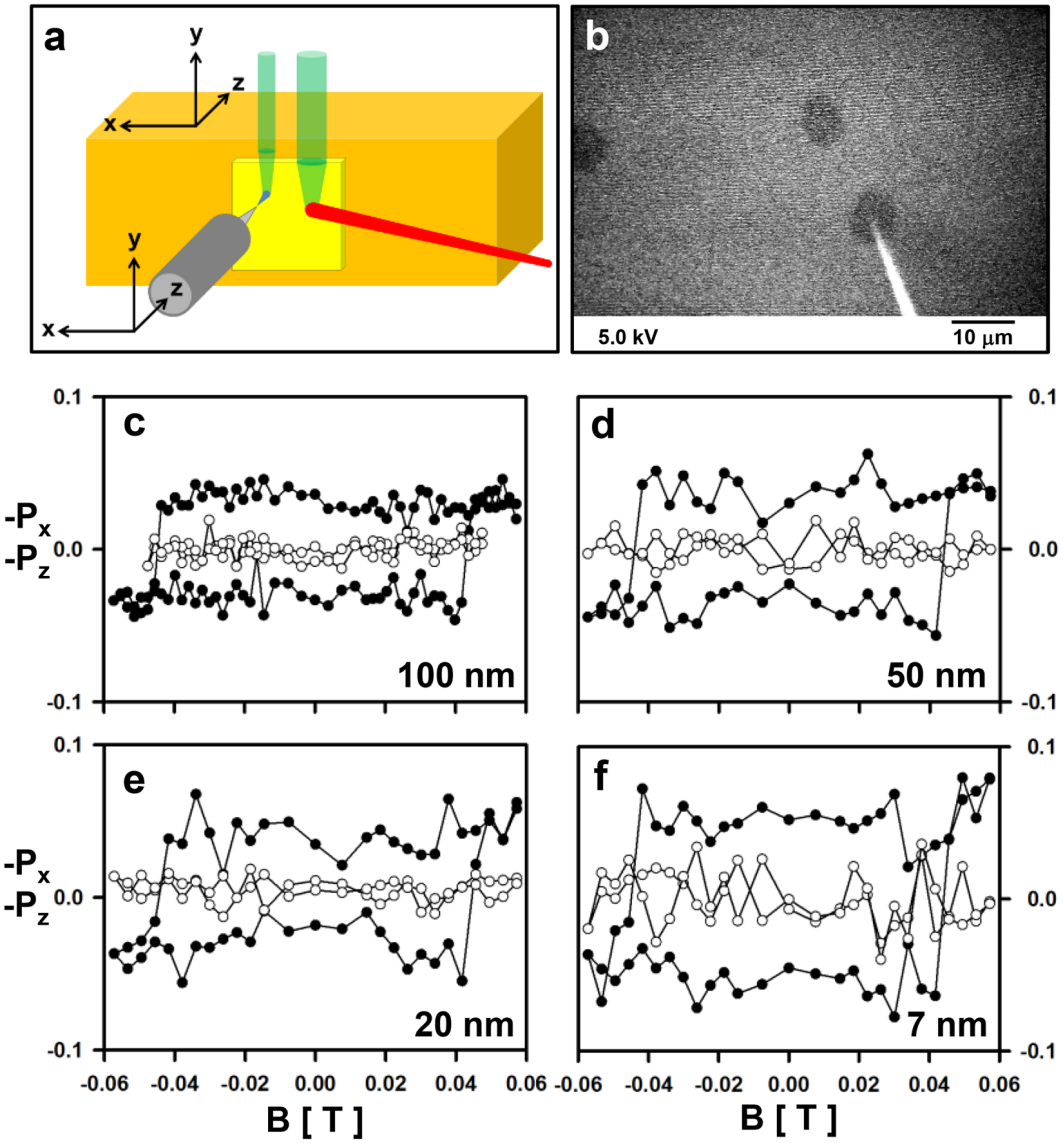
Spin polarisation as a function of source-drain distance. (**a**) A schematic view of the tip-target region. (**b**) Scanning electron microscopy micrograph of Fe dots (darker) deposited on a W (110) surface. (brighter). The W tip (bright) pointing towards one of the dots is also shown. Primary energy: 5 keV. The bar corresponds to a length of 10 µm. (**c**–**f**) −*P*_*x*_(*B*) (full circles) and −*P*_*z*_(*B*) (open circles) measured in the Fowler-Nordheim regime of STM at a fixed location above the Fe dot pinpointed in (**b**). (**c**) d = 100 nm, primary energy: 62 eV, field emission current: approximately 10 nA. (**d**) d = 50 nm, primary energy: 61.5 eV, field emission current: approximately 7 nA. (**e**) d = 20 nm, primary energy: 58 eV, field emission current: approximately 10 nA. (**f**) d = 7 nm, primary energy: 49 eV, field emission current: approximately 35 nA.

In a further set of experiments, we test the spin performance as a function of the source-drain distance. The Fe film is prepared by mask deposition as Fe dots with variable lateral size and a nominal thickness of 18 ALs. Figure 3a shows a schematic view of the tip-target region. The tip (attached to the grey cylinder) points towards the target (yellow). The electron beam from the JEOL gun is represented in red and the backscattered electrons in green. Figure 3b shows a scanning electron micrograph obtained by scanning the “red” electron beam across the target and in the presence of the tip. The tip-apex pointing towards one Fe dot (dark) is clearly visible (bright). The tip is then guided by STM towards the Fe dot pinpointed in Fig. 3b, and a set of hysteresis curves is measured at different tip-target distances (Fig. 3c–f). Except for the different amounts of noise affecting individual loops, we observe no significant change of the hysteresis loops upon changing from *d* = 100 nm (Fig. 3c) to the minimum recorded distance of 7 nm (Fig. 3f), where the spin polarisation is still substantial.

**Figure 4:**
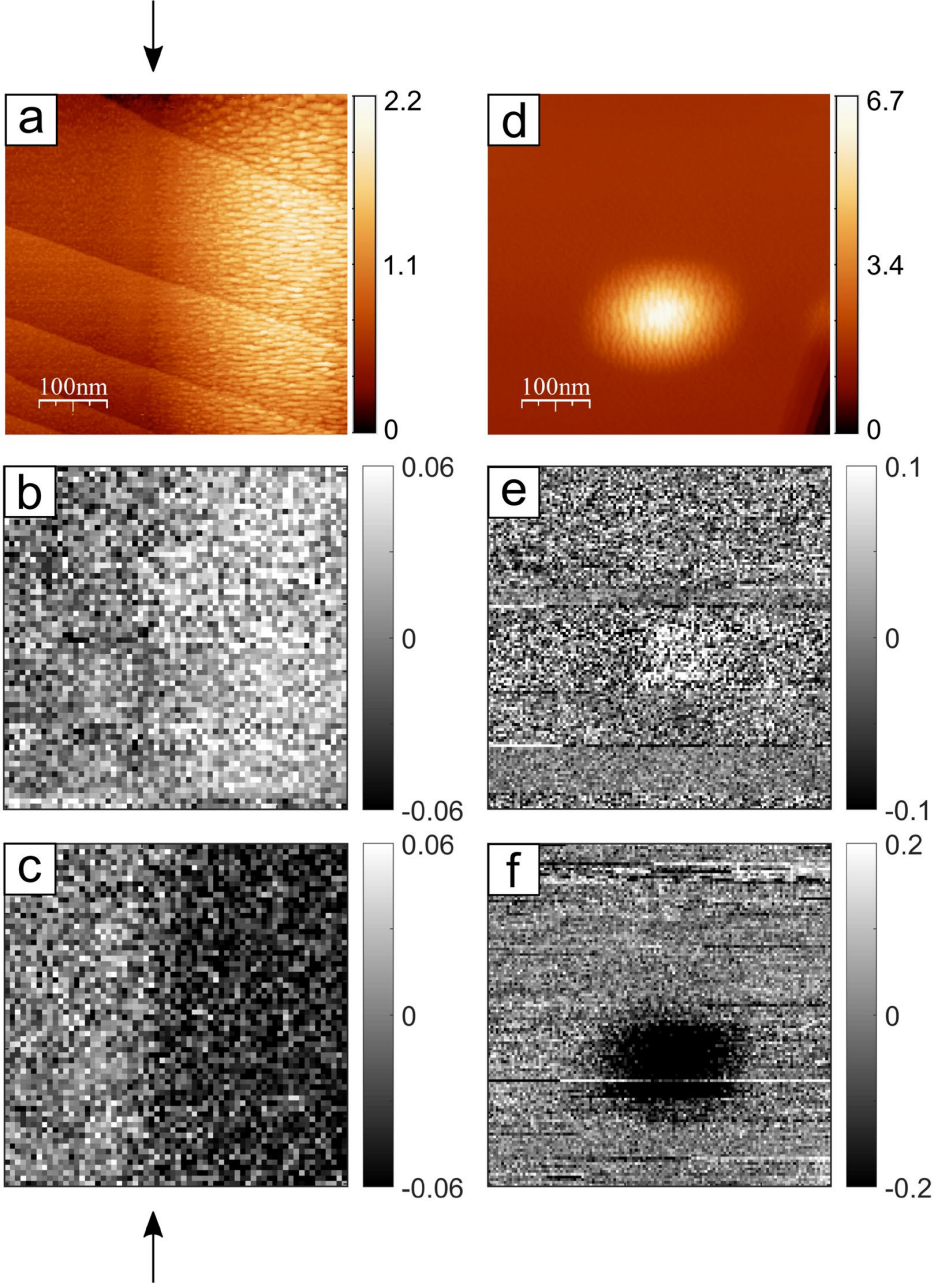
Magnetic imaging with spin-polarised backscattered electrons. (**a**) STM image (512 × 512 pixels) of the crossover region between an Fe-covered (right) and an Fe-uncovered (left) portion of the W (110) surface. An arrow indicates where the Fe film approximately starts: its thickness grows wedge-like when moving towards the right-hand side of the image (1 AL corresponds to a thickness of approximately 0.18 nm). The *z*-direction corrugation (colour coded in the vertical bar) is given in nm. (**b**,**c**) Images (64 × 64 pixels) of the −*P*_*x*_ component within the same field of view of (**a**), obtained after applying a magnetic field pulse of strength 0.03 T along +*x* (**b**) and −*x* (**c**). The spin polarisation is encoded using a grey scale (vertical bar). The size of the images is 500 × 500 nm^2^. Tip-target distance: 12 nm, tip voltage: −46 V. The current absorbed by the target is approximately 250 nA. (**d**) STM image of an Fe dot (bright, 512 × 512 pixels) surrounded by a clean W (110) terrace. The *z*-direction corrugation (colour coded in the vertical bar) is given in nm. (**e**,**f**) Images (128 × 128 pixels) of the −*P*_*x*_ component within the same field of view of (**d**), obtained after applying a magnetic field pulse of strength 0.07 T along + *x* (**e**) and −*x* (**f**). The spin polarisation is encoded using a grey scale (vertical bar). The size of the images is 500 × 500 nm^2^. Tip-target distance: 25 nm, tip voltage: −42 V, absorbed current: approximately 140 nA. Note that small Fe dots generally require a higher magnetic field pulse for switching than the continuous films.

We now report on the magnetic imaging^30^ experiments using the spin polarisation of backscattered electrons (the evidence of magnetic contrast was absent in the previous two^16,17^ attempts at measuring the spin polarisation of backscattered electrons). Figure 4a shows an STM image of the topography of the crossover region between an Fe-covered (right) and an Fe-uncovered (left) section of a W (110) surface. The borderline separating the two sections is indicated by an arrow. The colour code used to represent the *z*-corrugation is given as a vertical bar. The same region is then imaged in the backscattered electrons spin polarisation mode. The −*P*_*x*_ images are shown in Fig. 4b (after the application of a magnetic field pulse along+*x*) and 4c (magnetic field pulse applied along −*x*). The spin polarisation is encoded using a grey scale (vertical bar). The Fe-covered section is observed to change from bright (4b, right) to dark (4c right) upon switching the magnetic field. A similar magnetic contrast is recorded for an Fe-dot – STM topography in Fig. 4d and the −*P*_*x*_ images presented in Fig. 4e and f. We note that the spin polarisation measured in the positive magnetic field pulse channel (e) is not the same as the one measured in the negative (f) magnetic field pulse channel. This asymmetry provides the evidence of topographic cross-talk into the magnetic image that spoiled the detection of spin polarisation in an early magnetic imaging attempt^17^ (see the Methods section). In fact, when the tip is translated from the Fe-covered dot to the surrounding Fe-uncovered W terrace, the electron yield changes substantially^8^. If the two detectors compared for obtaining the value of the spin polarisation (see the Methods section) have a different response, they are bound to register this change of yield in addition to the change originating from the magnetic contrast. When the magnetic field is reversed (this degree of freedom was missing in ref.^17^), the non-magnetic contrast provides an asymmetric reading of the spin polarisation. The recording of an apparently “wrong” spin polarisation reading becomes therefore, in this context, a further essential step validating the finding of a finite spin polarisation in the backscattered spin system. When the entire hysteresis loop is measured, the pure magnetic signal can be extracted, as demonstrated in Figs 2 and 3 (see further comments in the Methods section).

We conclude by discussing the practical significance of a Fowler-Nordheim STM with spin polarisation analysis.

First, in a conventional Mott spin junction, the source ejects polarised electrons, and the target magnetisation acts to produce an asymmetry of the *transmitted* electron intensities. In this sense, the magnetisation of the target acts as a spin detector. In a one-magnet-only Mott spin junction, the spin detection is physically separated from the target, providing the practical advantage that the spin information can be obtained by addressing the magnetic state of the target only once. Furthermore, the non-magnetic primary beam is not affected by the external magnetic fields; thus, one can arbitrarily change the magnetic state of the target without affecting the detection process.

Second, $\overrightarrow{P}$ specifies the *direction* of the *local* magnetisation vector. Accordingly, the shape of the hysteresis curve in a given direction can be used to extract information about the magnetic material properties, such as the strength of the local magnetic anisotropies or the coercivity of bistable nanostructures and their evolution with temperature. Moreover, we expect this spin-polarised technology to find its main application in the imaging *and* characterisation of non-collinear spin textures, such as spin helices, skyrmions and domain walls. The actual state-of-the-art spatial resolution is set by Fig. 4. We observe that the size of the Fe dot in Fig. 4d (STM imaging) and Fig. 4f (spin-polarised Fowler-Nordheim imaging) are comparable. Because of the low efficiency of a spin polarimeter^20^, however, spin-polarised images are limited to fewer pixels than STM images. The spatial resolution is therefore set by the distance between the pixels, which is 5 nm in Fig. 4f. We anticipate that improving the efficiency of the spin polarimeter and optimizing the electron optical parameters will substantially (by at least a factor of 10, see ref.^31^) increase the number of pixels comprising a spin-polarised image and push the spatial resolution towards the 1 nm limit observed recently in non-spin-polarised Fowler-Nordheim imaging^8^.

Third, the appearance of a *P*_*x*_ component in the electron system of backscattered electrons, given a non-spin-polarised primary beam, must be compensated in steady-state operation of the junction by an opposite component appearing in the transmitted electron spin system and/or inside that region of the magnetic layer involved in the scattering process^7,32^. The very narrow channel defined in the magnetic layer by the size of the primary beam (in ref.^8^, as small as 1 × 1 nm^2^) sustains current densities that exceed the lower limit required for the onset of a magnetic instability^32^ so that the deviation from the equilibrium magnetsiation within this channel should become observable in steady-state operation.

Fourth, a *complete* spin-in – spin-out experiment along the lines of ref.^7^ but spatially localized that involves a source of spin-polarised electrons^10,13^, detection of current asymmetries *and* detection of the spin polarisation of the backscattered electrons^7^ is now conceivable.

These results are primarily a demonstration of a novel technology, and most of the processes discussed above have not yet been discussed theoretically. For instance, although the decrease of the magnitude of *P*_*x*_ at lower primary energies is in accordance with recent Monte Carlo simulations^33^, a quantitative estimate of the magnitude of the spin polarisation in terms of material parameters is lacking due to the lack of understanding of the elastic and inelastic exchange scattering processes and their dependence on the various kinematic parameters in the range of low energies covered by the Fowler-Nordheim regime. Whether the spin exchange scattering processes (in addition to transferring spin angular momentum to the backscattered electrons) can produce a net out-of-equilibrium spin accumulation on the magnetic target in the steady state of current operation and possibly after the current is removed also remains to be evaluated. A further issue specific to this technology is the actual number of electrons escaping the junction and whether their original spin polarisation is conserved. These questions entail modelling the transport of the excited electrons *i*. emerging within a nm-size spot, *ii*. crossing a region of intense (approximately 4 V/nm) and spatially inhomogeneous electric fields and *iii*. travelling over macroscopic distances (notably, mm) to reach the spin polarimeter. This research effort involves a multiscale approach that is currently under consideration.

## Methods

The present experiment uses a pair of Helmholtz coils that produce an external magnetic field pulse of variable strength (up to ≈ 10^−1^ T) along the positive and negative *x*-directions (Fig. 3a) with a controlled time structure. This is the key technical feature introduced to eliminate any instrumental asymmetry not related to the sought-for spin polarisation and with it the topographic cross-talk that rendered a previous study^17^ inconclusive. In fact, the Mott spin polarimeter detects the *P*_*x*_ (*P*_*z*_) values of an electron ensemble by collecting the electrons along one direction (*y* in the present configuration), letting these electron scatter from a target and measuring the number of electrons backscattered along two directions in the *yz* (*xy*) plane at angles ±*ϕ* with respect to the direction of the incident electron beam (*y*). The angle *ϕ* is typically chosen to be larger than 90° for maximizing spin sensitivity^20^, although in the backscattering geometry, the counting rate is reduced by a factor of ≈10^4^ with respect to the incoming current. The “left-right asymmetry” in the counting rates *N*(±*ϕ*) is related to the component *P* of the spin polarisation vector perpendicular to the scattering plane by the equation

1 $$\frac{N(+\varphi )-N(-\varphi )}{N(+\varphi )+N(-\varphi )}=P\cdot S,$$

where *S* is the Sherman function that describes the spin-dependent part of the scattering cross-section between the incoming electrons and the atoms comprising the scattering target^20,21^. *S* depends on the energy of the electrons, the scattering angle and on the atoms comprising the target within the Mott polarimeter. Since the spin-orbit coupling interaction between the incoming electrons and the target atoms is the origin of the left-right asymmetry, one typically choses a target consisting of “heavy” atoms (in the present case, Au) to maximize the spin asymmetry *P*·*S* for a given spin polarisation *P*.

In the present Mott polarimeter, we estimate that *S* ≈ 0.15^21^ for a scattering angle *ϕ* = 120° and an energy of ≈45 keV. The most important drawback of Mott spin polarimetry is the occurrence of unavoidable instrumental asymmetries – e.g., a slightly different efficiency of the detectors that are not related to spin polarization of the incoming beam but would produce a fictitious finite *P* when the above equation is used, or a contrast within a polarisation image that is not of magnetic origin^17^. In practice, instrumental asymmetries are conveniently taken into account using a slightly modified relation between left-right counting rates and spin polarisation that contains an “instrumental” parameter *Q* ^20^,

2 $$\frac{Q\cdot N(+\varphi )-N(-\varphi )}{Q\cdot N(+\varphi )+N(-\varphi )}=P\cdot S.$$

An unambiguous proof of finite spin polarisation *P* requires the knowledge of *Q* under exactly the same experimental conditions used to measure *P*. One possible strategy for determining *Q* is to measure *N*(±*ϕ*) using a beam of known spin polarisation^20^. In the present experiment, we have implemented the task of determining *Q* by using the extra degree of freedom provided by the variable external magnetic field. In a ferromagnetic sample, the magnetisation component *M* along the easy magnetisation axis as a function of the applied magnetic field *B* follows a hysteresis loop, meaning that the graph of *M*(*B*) has a closed topology with a special symmetry. Let us now define an operation <*M*> |_*B*_, consisting of averaging *M* over all the values of the magnetic field required to cover the entire hysteresis loop. Then, <*M*> |_*B*_ ≡ 0. Translated to the spin polarisation of the backscattered electron beam, this symmetry defines a state of averaged zero spin polarisation that allows to find *Q* simultaneously with the measurement of *P*(*B*) by means of the equation *Q*· < *N*(+*ϕ*) > |_*B*_− < *N*(−*ϕ*) > |_*B*_ = 0. The averaging operation can be implemented online while the counting rates are registered. Note that the need to run through the entire hysteresis loop is apparently in conflict with the need to detect the backscattered electron in an exactly vanishing magnetic field. In fact, a magnetic field at the site of the junction would inevitably bend the electrons away from the *y*-direction due to the effect of the Lorentz force: the counting rate almost vanishes and can be recovered only by a lengthy readjustment process of the entire electronic-optical system.

The externally applied magnetic field must have a time structure, defined in such a way that the field is exactly vanishing at the moment of backscattered electron generation and detection. In addition, magnetic fields as high as several 10^−1^ T are required to drive the Fe film through a complete hysteresis curve and to ensure both saturation and reversal of the magnetisation^27^. Such magnetic fields require comparatively high currents that can be kept active only for short times to avoid Joule heating and deterioration of the ultra-high-vacuum conditions. Accordingly, in a typical measurement sequence, the coils are loaded with a short current pulse that reaches its maximum after a certain, variable “load time” (typically in the microsecond range) at which the maximum magnetic field *B* is produced. *B* drives the film into a magnetic state *M*(*B*). The current is then switched off so that by engineering the electric circuit, one can achieve a rapid, almost linear-in-time decrease of the current to exactly zero^28^. At that point in time, one proceeds with the detection of the backscattered counting rates either using SEMPA or in the Fowler-Nordheim regime of STM. Provided that the hysteresis loop of the target is a square (as is the case for Fe/W (110)), the magnetic state resulting after the field pulse has been switched off retains some remanent finite magnetization that should not be excessively different from the *M*(*B*) state achieved with finite *B*.

We note that despite the field-free detection, the key symmetry <*P*> |_*B*_ = 0 is conserved.

When spin-polarised imaging is attempted, the primary beam is scanned over a certain range of the *xy* plane, and one is confronted with an instrumental asymmetry that in principle depends on the (*x,y*) coordinates: *Q* = *Q*(*x, y*). The *xy* dependence arises because the spatially dependent backscattered electron yield triggers a non-linear detector response and/or because the backscattered electrons have a variable spatial origin, thus effectively introducing a spin polarisation of the scattered electrons with the non-magnetic origin via spin-orbit coupling. Through these mechanisms, one often observes details of the topography in the polarisation image – the topographic feedthrough that introduces uncertainties in the assignment of any contrast detected in the polarisation image. If the spatial origin of the backscattered electrons is kept constant while the hysteresis curve is measured (Figs 2 and 3), we can eliminate instrumental asymmetries by averaging over the magnetic field. Instead, during a xy scan, *Q* depends on (*x,y*), and the elimination of instrumental asymmetries cannot be exactly performed at each location.

The spin analysis channel in the present experiment is unconventional in the sense that the backscattered electrons are conveyed towards the Mott target along a straight path (parallel to the *y*-direction), indicating that independently of their energy, all electrons are in principle accepted within the spin detector. By contrast, in conventional SEMPA, the electron optical path is arranged to accept only the relatively low-energy backscattered electrons (kinetic energy of approximately 6 eV), which are not only the most intensive but also have the largest spin polarsiation for a given target magnetisation state^25,26^ (in the present system, the spin polarisation in energy-selected SEMPA is approximately 40%^27^). Because all electrons are allowed into the spin detector, including those that are less^25,26^ spin polarised, we expect (and indeed observe) our energy-unselected SEMPA to measure a reduced spin polarisation.

### Data Availability.

Data are available from the ETH Zurich Data Archive: http://doi.org/10.3929/ethz-b-000188637.

## Electronic supplementary material

Recording of the real-time measurement of a hysteresis loop in the Fowler-Nordheim regime of STM
